# Microwave Hydrothermal Carbonization of Rice Straw: Optimization of Process Parameters and Upgrading of Chemical, Fuel, Structural and Thermal Properties

**DOI:** 10.3390/ma12030403

**Published:** 2019-01-28

**Authors:** Sabzoi Nizamuddin, Sundus Saeed Qureshi, Humair Ahmed Baloch, Muhammad Tahir Hussain Siddiqui, Pooja Takkalkar, Nabisab Mujawar Mubarak, Deepa K. Dumbre, Gregory J. Griffin, Srinivasan Madapusi, Akshat Tanksale

**Affiliations:** 1 School of Engineering, RMIT University, Melbourne 3000, Australia; humairbaloch@hotmail.com (H.A.B.); tahirchemical@gmail.com (M.T.H.S.); Pooja.jadhav676@gmail.com (P.T.); deepadumbre@gmail.com (D.K.D.); gregory.griffin@rmit.edu.au (G.J.G.); nd08ch248@yahoo.com (S.M.); 2 Institute of Environmental Engineering and Management, Mehran University of Engineering and Technology, Jamshoro 76090, Sindh, Pakistan; sundussaeed11te95@gmail.com; 3 Department of Chemical Engineering, Faculty of Engineering and Science, Curtin University, 98009 Sarawak, Malaysia; 4 Department of Chemical Engineering, Monash University, Clayton 3800, Australia; agbaloch0@gmail.com

**Keywords:** rice straw, hydrochar, microwave-induced hydrothermal carbonization, energy properties

## Abstract

The process parameters of microwave-induced hydrothermal carbonization (MIHTC) play an important role on the hydrothermal chars (hydrochar) yield. The effect of reaction temperature, reaction time, particle size and biomass to water ratio was optimized for hydrochar yield by modeling using the central composite design (CCD). Further, the rice straw and hydrochar at optimum conditions have been characterized for energy, chemical, structural and thermal properties. The optimum condition for hydrochar synthesis was found to be at a 180 °C reaction temperature, a 20 min reaction time, a 1:15 weight per volume (w/v) biomass to water ratio and a 3 mm particle size, yielding 57.9% of hydrochar. The higher heating value (HHV), carbon content and fixed carbon values increased from 12.3 MJ/kg, 37.19% and 14.37% for rice straw to 17.6 MJ/kg, 48.8% and 35.4% for hydrochar. The porosity, crystallinity and thermal stability of the hydrochar were improved remarkably compared to rice straw after MIHTC. Two characteristic peaks from XRD were observed at 2θ of 15° and 26°, whereas DTG peaks were observed at 50–150 °C and 300–350 °C for both the materials. Based on the results, it can be suggested that the hydrochar could be potentially used for adsorption, carbon sequestration, energy and agriculture applications.

## 1. Introduction

The rice industry produces a large amount of agricultural waste in the form of rice straw. According to the Food and Agricultural Organization of the United Nations, annual rice production is 600 million tonnes worldwide [1]. Kadam et al. [2] suggested that each ton of grain harvested leaves 1.35 tonnes of rice straw in the field. Hence, annual global production of rice straw is of the order of 650–975 million tonnes [3]. This rice straw is usually either burnt in the open atmosphere or dumped into landfills. Both disposal techniques are environmentally hazardous methods. Therefore, it is desirable to utilize a better method for utilizing this material. The utilization of rice straw may serve a double purpose—the removal of an unwanted residue while producing fuels or chemicals. In terms of biofuels, rice straw can be converted into three phases of biofules, i.e., gaseous (biogas), liquid (bio-oil) and solid (char) products. 

Char is a carbonaceous material formed by the thermal decomposition of biomass- or waste-derived carbons [4]. Char possesses a great potential to be utilized for a vast range of applications including the production of biomethane in anaerobic digestion, catalysis, water purification, gas storage and soil amendment, etc. [5,6,7,8,9]. Chars are of two types based on their synthesis method: pyrolysis char (referred as biochar) and hydrothermal chars (termed as hydrochar) [10]. Hydrochar differs from biochar in terms of the production method and the presence or absence of a bulk water phase during synthesis; thus, the hydrochar synthesis process is called hydrothermal carbonization [11]. Hydrothermal carbonization is a thermochemical decomposition process in which biomass is heated in water at modest temperatures (180–350 °C) and pressures (2–10 MPa) to produce hydrochar as a main product and water-soluble organic substances as by-products [12]. Hydrothermal carbonization can be divided into two types (conventional and microwave hydrothermal carbonization) depending on the energy source for heating. In conventional hydrothermal carbonization processes, heat is transferred externally by radiation, convection or conduction. However, conventional heating methods have certain shortcomings such as high heat loss to the surroundings, high heat transfer resistance and damage to reactor walls as the walls are typically exposed to a much higher temperature [13]. In the microwave-induced hydrothermal carbonization (MIHTC), the biomass is heated through microwaves, unlike conventional hydrothermal carbonization where heat is transferred through a temperature gradient, i.e., conduction or convection [14]. Therefore, it is suggested that the MIHTC is a faster method with homogenous temperature distribution through the target biomass which reduces processing time and cost significantly.

MIHTC is still an emerging technique but has been used for the carbonization of lignocellulosic biomass, human waste and seafood waste [14,15,16,17,18,19,20,21,22]. Guiotoku et al. [20] was the first to utilize microwave heating for hydrothermal carbonization (HTC) processes and studied the MIHTC of pine saw dust and α-cellulose. The moisture content present in the biomass and distilled water used in the reaction was favorable for the MIHTC process because the water molecules that readily couple with the electromagnetic field result in microwave dielectric heating [14]. Different studies are conducted through the MIHTC of various biomass sources including α-cellulose and pine sawdust [20], rapeseed husk [21], glucose [16], prosopisafricana shell [17], human faecal sludge and sewage sludge [22]. Guiotoku et al. [20] studied the MIHTC of two different lignocellulosic biomass and inspected the effects of reaction time on the yield of hydrochar. Another study conducted by Elaigwu and Greenway [21] investigated the effect of two parameters of MIHTC, i.e., temperature and time on hydrochar yield from the MIHTC of rapeseed husk. Elaigwu and Greenway [17] conducted a comparative study for HTC using both conventional and microwave heating systems and examined the effect of the reaction temperature on the hydrochar yield of prosopisafricana shells with two different heating systems. Nizamuddin et al. [23] upgraded the different properties of rice husk by using the MIHTC process. They also studied the effect of four process parameters of MIHTC on the hydrochar yield as well observed a significant improvement in the chemical, fuel, structural and thermal properties of hydrochar after the MIHTC process. A comparative review on MIHTC and microwave pyrolysis was published recently [24]. 

In this study, the process parameters of MIHTC have been optimized for maximum hydrochar yield by using the central composite design (CCD). The parameters include reaction temperature (180–220 °C), reaction time (20–60 min), biomass to water ratio (1:5–1:15 w/v) and biomass particle size (1–3 mm). Further, the rice straw and optimized hydrochar product are characterized for higher heating value (HHV), ultimate analysis and proximate analysis. Moreover, Fourier transform infrared (FTIR) analysis, scanning electron microscopy (SEM), X-ray diffraction (XRD) analysis and thermogravimetric (TGA) analysis is carried out for both the rice straw and optimized hydrochar. Additionally, N_2_ adsorption isotherms for the rice straw and optimized hydrochar have been plotted.

## 2. Materials and Methods

### 2.1. Rice Straw

Rice straw was received from Dowens Rice Hulls Pty. Ltd., Victoria, Australia and was washed with tap water twice and then was washed with deionized water in order to eliminate the dirt and the surface impurities. Then, the double-washed rice straw was dried in an oven at 105 °C for 24 h. The rice straw was ground to particle sizes between 1–3 mm using a MF 10basic, IKA Labortechnik crusher in order to investigate the effect of particle size on the hydrochar yield. The general lignocellulosic composition of rice straw is 32% cellulose, 35.7% hemicellulose, 22.3% lignin and 10% extractives [25].

### 2.2. Microwave-Assisted Hydrothermal Carbonization Process

**Figure 1:**
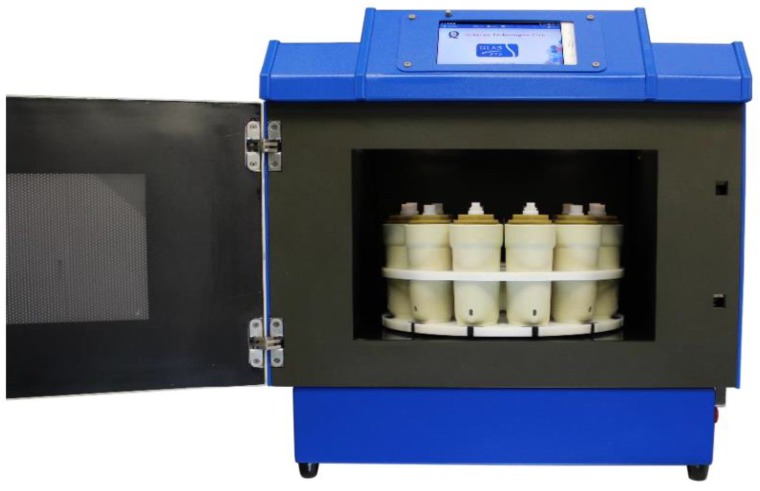
The QLab pro microwave digestion system.

A QLab pro microwave digestion system, Questron Technologies Corp., with a maximum power of 1200 W and temperature of 230 °C as shown in Figure 1 was used to conduct the experiments for the MIHTC of rice straw. First, the deionized water and rice straw were put in the reactor vessel at the desired biomass to water ratio, and then the vessel was tightly sealed. Then, the process parameter values were set for the experimental run followed by the reaction of the samples. After the completion of the reaction, the reactor was fan-cooled down to room temperature. The cooling fan helps to maintain the temperature inside the reactor during the operation, and it supports in cooling down the reactor vessel once the reaction is completed as the heater will be auto-turned off. As the reactor was cooled down to room temperature, the gaseous products, which mainly consists of carbon dioxide [26] (not investigated in this study) were vented out, and the hydrochar and bio-oil were separated by filtration. The final hydrochar product was washed several times with deionized water and then was dried in an oven at 105 °C for 24 h.

### 2.3. Hydrochar Yield (%) 

The hydrochar yield (wt %) is determined as the mass ratio between the hydrochar and the raw rice straw loaded into the reactor as shown in Equation (1).

Yield (%) of hydrochar = (Weight_dh_/Weight_drs_) × 100 (1)

where Weight_dh_ is the amount of hydrochar product and Weight_drs_ is the amount of rice straw loaded in the reactor.

### 2.4. Optimization of Process Parameters Using CCD

In this study, the CCD of the response surface methodology was utilized in order to optimize the process parameters of the MIHTC of rice straw for the optimum synthesis of hydrochar. Table 1 shows the process variables and their values for the hydrochar synthesis. The experimental design suggested 25 runs for the synthesis of hydrochar. Further, the design expert software version 6.08 and company stat-ease, Inc. 2000 (Minneapolis, United States) was used to find the optimal reaction conditions for hydrochar synthesis within the range of parameters tested. A complete design matrix with responses (hydrochar yield wt %) is listed in Table 2.

### 2.5. Characterization of Rice Straw and Optimized Hydrochar

The CHNS/O analysis of rice straw and hydrochar was carried out using a 2400 Series II CHNS/O Elemental Analysis (Perkin Elmer). The oxygen percentage was calculated by the mass balance after subtracting the masses of hydrogen, carbon, nitrogen, sulphur and ash. HHV was calculated using Equation (2) proposed by the Boie’s equation [27], and the energy densification was calculated by Equation (3).

HHV (MJ/kg) = 0.3516(C) + 1.16225(H) − 0.1109(O) + 0.628(N)(2)

where C, H, O and N represent the mass contents of carbon, hydrogen, oxygen and nitrogen.

Energy densification = HHV_dh_/HHV_drs_ (3)

The proximate analysis of both the feedstock and main product was conducted by using TGA following the method of Manoj et al. [28]. A Perkin Elmer FTIR Spectrometer (TA 8000) was used to study the FTIR spectra (4000–550 cm^−1^) of rice straw and hydrochar using Attenuated total reflectance (ATR) mode. The SEM graphs were obtained by using a Philips XL30 SEM (Edwards High Vacuum, Sussex, UK). The TGA/DTG analysis was studied at 50–900 °C in nitrogen at 20 mL/min flow using a Perkin Elmer STA 6000. The sample (approximately 10 mg) was placed in a crucible, the temperature was increased at 10 °C/min and the readings were recorded every 30 s.

## 3. Results and Discussion

### 3.1. Optimization of Process Parameters for Hydrochar Synthesis

The MIHTC is strongly influenced by the process parameters; this research emphasizes the optimization of the hydrochar yield across the process parameters of MIHTC. Several experiments were conducted for the optimization of the process parameters based on a design expert software (6.08, State-Ease, Inc. 2000, Minneapolis, MN, United States). The parameters used were (with their ranges) reaction temperature (180–220 °C), reaction time (20–60 min), particle size (1–3 mm) and biomass to water ratio (1:5–1:15 w/v), and the results obtained were employed in the design analysis to obtain the optimized yield of hydrochar. Analysis of variance (ANOVA) was used to analyse the experimental results as presented in Table 3. The regression model was evaluated based on a higher Fisher (F) value and a lower *p* value. The high F value of 396.0004 and low probability *p* value of <0.0001 were obtained for the model of the data, which implies the model was relevant. The equation developed on the effect of the parameter levels on the hydrochar yield is shown in Equation (4).

(4) $$\begin{array}{ll} \mathrm{Yield}=44.72264 & -8.36678\left(A\right)-1.27367\left(B\right)+0.101172\left(C\right)+0.123641\left(D\right)\\ & +0.175091\left(\mathrm{AB}\right)+0.127969\left(\mathrm{AC}\right)-0.16454\left(\mathrm{AD}\right)-0.22143\left(\mathrm{BC}\right)\\ & -0.12982\left(\mathrm{BD}\right)+0.010792\left(\mathrm{CD}\right)+2.55697\left(A^{2}\right)+0.130184\left(B^{2}\right)\\ & -0.26385\left(C^{2}\right)+0.457945\left(D^{2}\right) \end{array}$$

where A indicates the coded value of the reaction temperature, B represents the coded value of the reaction time, C denotes the coded value of the particle size and D defines the coded value of the biomass to water ratio. One-factor coefficients show the effect of any particular factor, while two-factor coefficients highlight the interactive effect between two factors. A positive (+) sign represents a synergistic effect, whereas a negative (−) sign illustrates an antagonistic effect.

**Figure 2:**
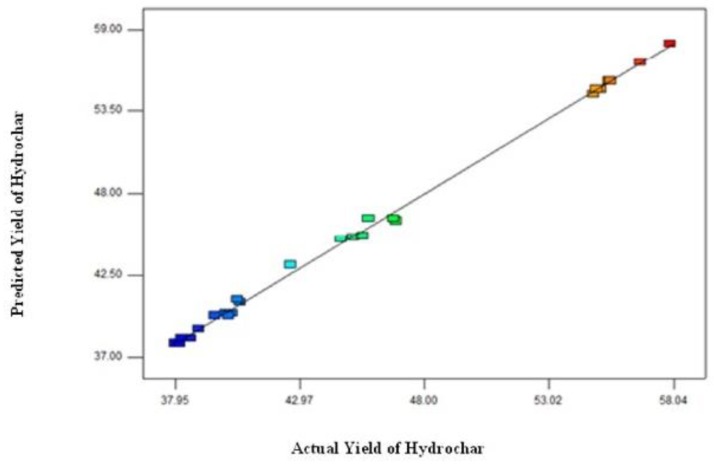
The relationship between the model and experimental values of the hydrochar yield.

Figure 2 shows theoretical (predicted) values versus the experimental (actual) values for the yield of hydrochar. It is apparent from Figure 2 that the predicted values of the hydrochar yield are close to the actual values of the hydrochar yield, which confirms that the established model demonstrates an effect to correlate the MIHTC parameters with the hydrochar yield.

**Figure 3:**
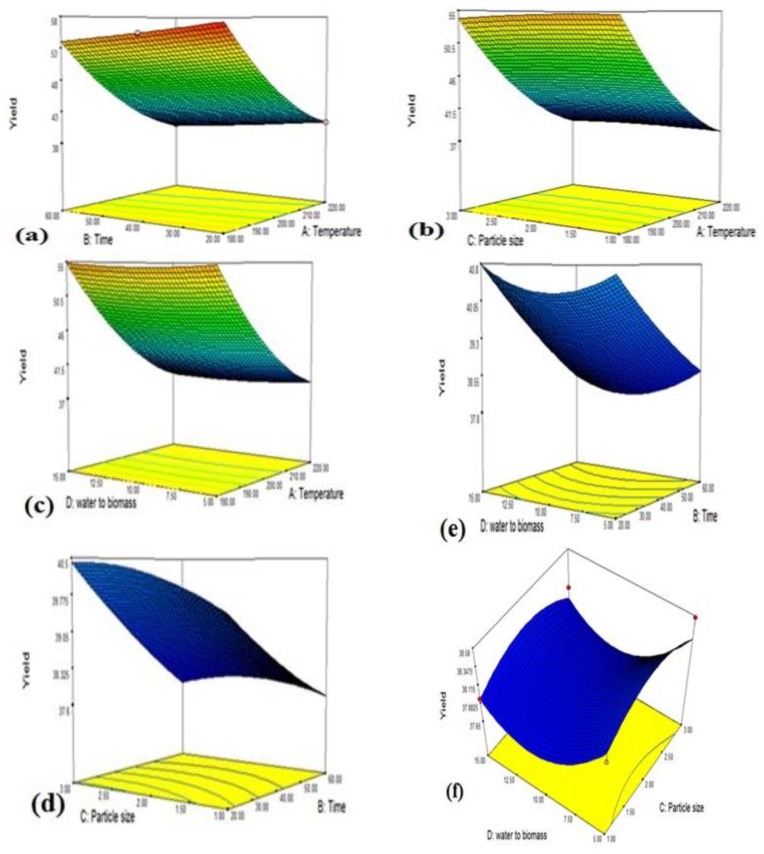
Three-dimensional graphs of (**a**) the reaction temperature and time, (**b**) the reaction temperature and particle size, (**c**) the reaction temperature and biomass to water ratio, (**d**) the reaction time and particle size, (**e**) the reaction time and biomass to water ratio, and (**f**) the particle size and biomass to water ratio.

The effect of the MIHTC parameters and their interaction on the yield of hydrochar is exhibited in the 3-dimensional response graphs shown in Figure 3a–f. The combined effect of the reaction time and temperature is presented in Figure 3a, which suggests that a higher yield of hydrochar will be produced at a lower reaction temperature and lower reaction time. Similar trends for the HTC of oil palm shells was observed in the literature [29]. Figure 3b illustrates the joint effect of temperature and particle size, and it is observed that a higher particle size and lower reaction temperature generate a greater hydrochar yield. The interaction between temperature and the biomass to water ratio is presented in Figure 3c, which reports that both a low temperature and low biomass to water ratio will produce a greater yield of hydrochar. The combined effect of the reaction time with the particle size is shown in Figure 3d. The graph shows that a low reaction time and high particle size will form a higher yield of hydrochar. Figure 3e demonstrates the combined effect of the reaction time with the biomass to water ratio. It can be seen from Figure 3e that a lower time and lower biomass to water ratio will generate a greater yield of hydrochar. Figure 3f shows the combined interactive effect of the biomass to water ratio and particle size. It is evident from Figure 3f that a higher particle size and lower biomass to water ratio will form a higher yield of hydrochar.

### 3.2. Characterization of Rice Straw and Optimized Hydrochar

#### 3.2.1. HHV and Energy Density

The HHV of the raw rice straw and optimized hydrochar is compared to various hydrochars synthesized by the MIHTC of different biomasses and of peat and lignite coals in Table 4. It is shown in Table 4 that the HHV of rice straw is 12.3 MJ/kg which increased to 17.8 MJ/kg in optimized hydrochar after the MIHTC. It is evident from Table 4 that the HHV of optimized hydrochar is similar to that of hydrochars produced from the microwave processing of various biomasses such as bamboo hydrochar (17.2 MJ/kg), barley straw hydrochar (17.8 MJ/kg), wheat straw hydrochar (17.9 MJ/kg) and rapeseed husk hydrochar (21.6 MJ/kg). Further, it is comparable to lignite coal (16.9 MJ/kg) and peat (15.4 MJ/kg), confirming the suitability of hydrochar as a solid fuel.

An increased HHV of the optimized hydrochar after MIHTC is credited mainly to the influence of the reaction temperature and reaction time of the MIHTC process. Higher reaction temperatures cause an enhancement in the carbon content resulting in an improved HHV [33]. In addition to that, the HHV of hydrochars goes up at higher reaction times, which represents higher degrees of carbonization achieved during MIHTC [17]. It is reported that the HHV value of the hydrochar produced from the MIHTC of glucose increased from 16.90 MJ/kg at 15 min to 21.30 MJ/kg at 45 min and remained stable afterwards [16]. Other research found that the HHV of hydrochar increased from 18.25 MJ/kg to 21.10 MJ/kg by increasing the MIHTC reaction time from 60 min to 120 min [14]. The improvement in HHV of optimized hydrochars may be attributed to a higher lignin content of rice straw. The HHV of lignin is higher compared to hemicellulose and cellulose because of the degree of the oxidation. The lignin has a lower oxidation degree as compared to cellulose and hemicelluloses [34,35].

Energy densification defines the process of transforming feedstock with a lower energy density to fuels with a higher energy density. During the MIHTC of biomass, the energy density depends upon the higher degree of carbonization, i.e. reactions such as condensation, decarboxylation and dehydration, which increase the carbon content of the hydrochar [17]. The energy densification of the optimized hydrochar is 1.43, indicating the MIHTC process alters the lower energy rice straw into a higher energy hydrochar. The energy densification of the optimized hydrochar, i.e., 1.43, is in the range of the hydrochars produced from MIHTC of different biomasses reported in literature (1.23–1.55) [16].

An improvement in the energy density of the optimized hydrochar is attributed mainly to the reaction time and temperature of the MIHTC process. The energy densification improved by increasing the temperature [19]. Nakason et al. [36] investigated the effect of both the reaction time and temperature on the energy density of hydrochars produced from rice husk. It was observed that the energy densification increased from 1.07 to 1.20 by increasing the reaction temperature from 140 °C to 200 °C, whereas it increased from 1.16 to 1.20 by increasing the reaction time from 1 hour to 4 h. 

#### 3.2.2. Ultimate Analysis

The CHNS/O analysis describes the elemental composition (carbon, hydrogen, oxygen, nitrogen and sulphur) of a material [35]. The ultimate analysis defines the composition and may be used to determine the likely amount of gas released by combustion as well as the quantity of oxygen needed to burn fuels or biomass [37]. More oxygen and less carbon in biomass or biofuels are not favorable because the high oxygen content lowers the HHV, causes corrosion of the metallic reactor vessels and its joints and also increases the instability of biofuels [38].

**Figure 4:**
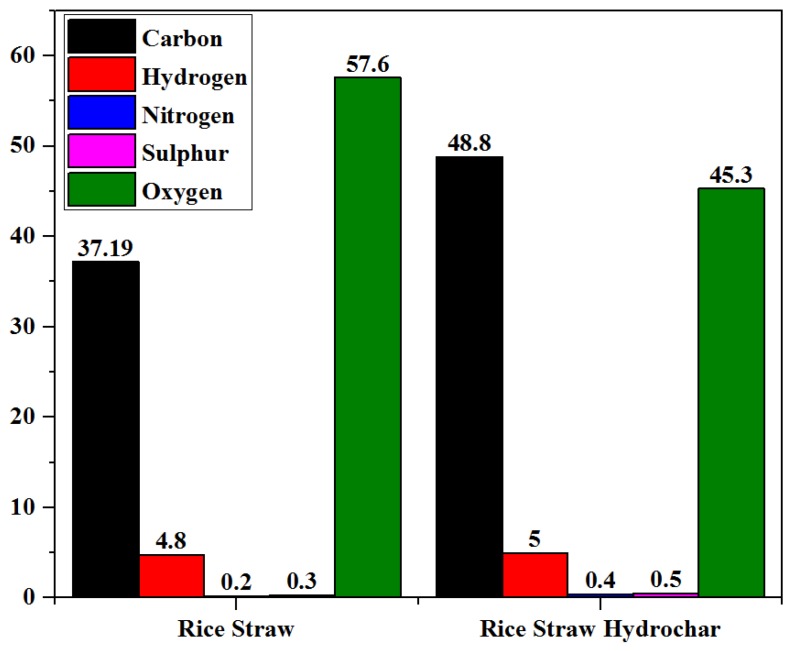
The ultimate analysis of rice straw and the optimized hydrochar.

The ultimate analysis of raw rice straw and the optimized hydrochar is shown in Figure 4. The carbon value of rice straw was 37.19%, which increased to 48.8% in an optimized hydrochar. On the other hand, the oxygen content of rice straw was 57.6% which decreased to 45.3% in the optimized hydrochar after the MIHTC process. Higher carbon and lower oxygen contents are considered to enhance the combustion properties of hydrochars [35,39]. An increase of the carbon content and decrease of the oxygen content after the MIHTC process are attributed to the deoxygenation reactions (decarboxylation and dehydration reaction) taking place during the MIHTC which is influenced by the process parameters (reaction temperature and reaction time) [40,41]. The higher temperature and time cause a greater increase in the carbon content and a decrease in the oxygen content [14,22]. Kannan et al. [14] reported that the carbon content increased from 37.0% to 49.0% whereas oxygen percentage reduced from 50.67% to 39.31% by varying the reaction time from 60 min to 120 min. Another research work studied the MIHTC of fish waste and found that the carbon content rose from 27.0% to 50.0% whereas the oxygen content decreased from 65.0% to 37.0% by increasing the reaction time from 60 min to 120 min [15].

A minor increase in the nitrogen of hydrochar products was observed after the MIHTC process. The nitrogen content of rice straw was 0.2%, which increased to 0.4% in the hydrochar, indicating that the nitrogen content in rice straw is not removed by dissolution in hot water and is retained in the hydrochar [42]. An increase in the nitrogen content of hydrochars could be attributed to the protein fraction being less reactive during the process.

**Figure 5:**
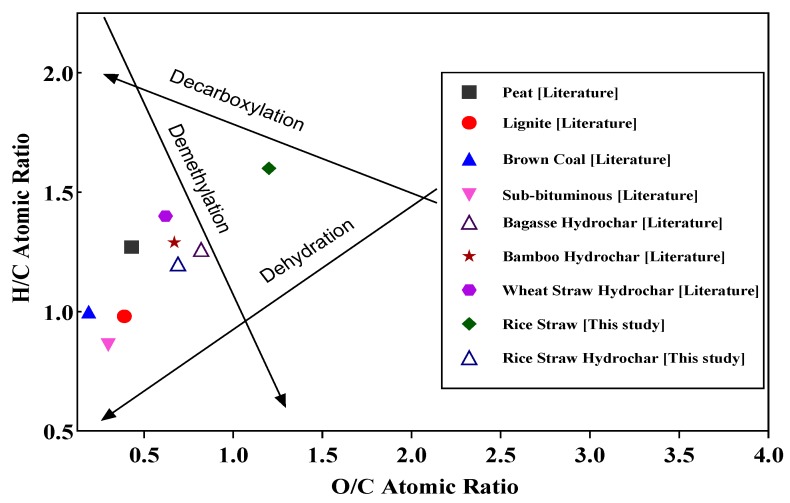
The H/C atomic ratio versus the O/C atomic ratio.

A Van Krevelen diagram is used to show the changes taking place in the ultimate analysis with the types of reactions during hydrocarbonization and to relate these changes to fuel quality [43,44]. The results of this study are compared to hydrochars produced from the MIHTC of different biomasses and various low-rank coals [45] in Figure 5. Figure 5 plots the H/C atomic ratio versus the O/C atomic ratio. Generally, a lower H/C and O/C value will result in a higher fuel quality. The results show that the H/C and O/C ratios were lower in the optimized hydrochar than in raw rice straw as a consequence of the carbonization reactions with a possible increase in the aromaticity of the hydrochar [46]. Further, the H/C and O/C values of the optimized hydrochar are similar to various hydrochars produced from MIHTC and comparable to different types of coal. The lower O/C ratio of the hydrochar showed the advantages of the HTC process over the conventional torrefaction [42]. It is reported in the literature that partially carbonized products having low H/C and O/C atomic ratios are produced from the HTC process [20].

Lower H/C and O/C atomic ratios of optimized hydrochars compared to raw rice straw (reference Figure 5) indicates that dehydration, demethylation and decarboxylation reactions occurred during the MIHTC [47]. 

#### 3.2.3. Proximate Analysis

The proximate analysis is an important characteristic of biomass and fuels which relates the energy content by comparing the ratio of combustible substances to noncombustibles. It measures the fixed carbon, moisture, volatile matter and ash contents present in the samples. The proximate analysis results of rice straw and its optimized hydrochar is listed in Table 5, and values are compared to those of various coals. Generally, fuel with low moisture, low volatiles and high fixed carbon is considered a good fuel because higher fixed carbon causes an increment in HHV whereas higher volatile matters present in fuel drops its combustion performance [48]. Higher amounts of volatile matter present in biomass has a significant effect on its direct combustion and co-combustion with coal causing a reduced combustion performance and higher pollutant emissions [49]. The rice straw hydrochar can be a suitable candidate for co-combustion with coal due to a less volatile matter in hydrochar; hence, it will provide better combustion properties and will emit fewer pollutants during co-combustion with coal. In addition to that, solid biofuels will improve the flame stability and will provide better control of the burning process [50].

From Table 5, the moisture content and volatile matter of rice straw were 8.53% and 70.20%, which decreased to 2.5% and 45.6% respectively in the optimized hydrochar. Further, the fixed carbon content of rice straw was 14.37% which increased to 35.4% for the optimized hydrochar. An increase in the fixed carbon and a decrease in the volatile matter are affected by the reaction temperature because biomass degrades significantly at higher temperatures resulting in a decreasing volatile matter and ultimately enhancing the fixed carbon content [48]. A rise in the value of fixed carbon is indicated by a release of volatile matters taking place during the MIHTC [51]. Nizamuddin et al. [23] observed an increased in fixed carbon after the MIHTC of rice husk and suggested that it is attributed to the release of the higher amount of volatile matters during the MHTC of rice husk. According to Yuan et al. [48], biomass is degraded significantly at higher temperatures, which results in the decrement of volatile matters, ultimately increasing the fixed carbon content. The same study reported the conversion of corn stalk into a good quality fuel because the volatile matter was decreased and the fixed carbon content was increased in the products. An increase in fixed carbon increases the HHV while the higher amount of volatile matters in fuel decreases its combustion performance.

The ash content increased from 6.9% of rice straw to 16.5% in rice straw hydrochar, which is in agreement to previous literature [26,52]. Reza et al. [53] observed that the ash content of rice hull was 21% which increased to 29.8% in rice hull biochar, which is attributed to the reaction temperature. The proximate analysis results of the optimized hydrochar are similar to those of various types of coal as shown in Table 5.

#### 3.2.4. FTIR Analysis

**Figure 6:**
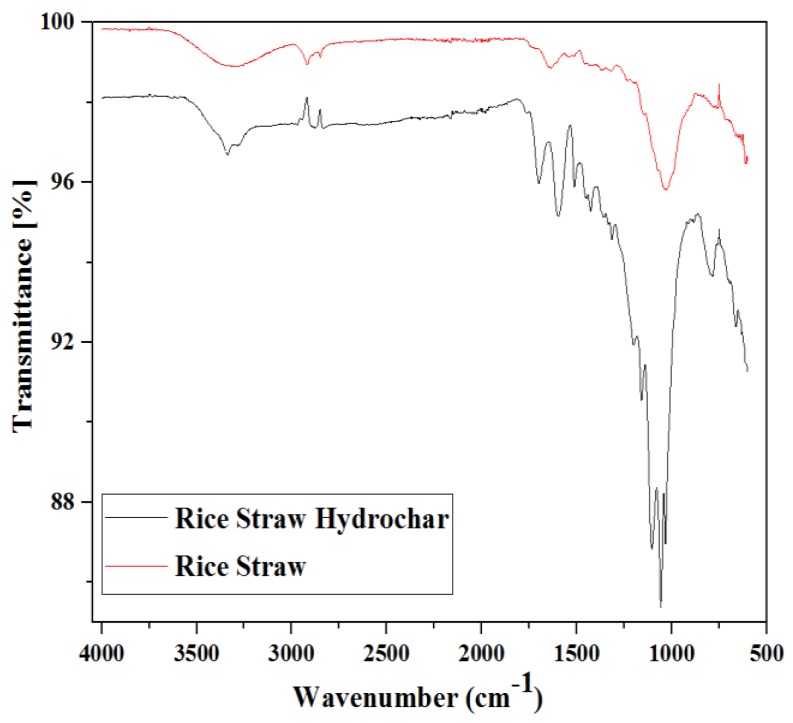
The Fourier transform infrared (FTIR) analysis of rice straw and optimized hydrochars.

The functional groups present on the hydrochar surfaces can influence biodegradability and play a vital role in adsorption processes. FTIR spectroscopy is conducted to identify the functional groups present on the surface of biomass and biofuels. It also indicates the surface changes occurring during the processing of biomass [58]. The FTIR analysis of both the rice straw and optimized hydrochars was conducted by using FTIR spectra at the wavenumber ranges of 4000 to 550 cm^−1^ as shown in Figure 6. The spectra of the found compounds offer information about the readily available bonds [59].

A strong and broadband peak was observed for both the rice straw and optimized hydrochar at around a 3400 to 3300 cm^−1^ –OH broad vibration of carboxylic, phenolic or alcoholic functional groups. This peak is decreased slightly in hydrochars suggesting that dehydration reactions are occurring during the MIHTC [60]. The peak around 2900–2800 cm^−1^ for both the rice straw and hydrochars shows the C–H stretching vibrations of the –CH_3_ and –CH_2_ functional groups representing volatile removal [61] and the CH_n_ stretching vibration which shows the presence of aliphatic and aromatic compounds. The bands at 1700 cm^−1^ are assigned to the carboxyl C–O groups [62]. The peaks at 1653 cm^−1^ and 1637cm^−1^ were observed for rice straw and hydrochar, which belongs to the C=N stretching due to the presence of oximes functional groups. The modes of aromatic C–H vibrations have been reported at 1417 cm^−1^ [63]. Several peaks were found in both the rice straw and the optimized hydrochar at 1100 cm^−1^ and 752 cm^−1^ indicating the existence of aromatic functional groups, respectively which did not change with thermochemical processing, i.e., MIHTC. 

#### 3.2.5. SEM Analysis

**Figure 7:**
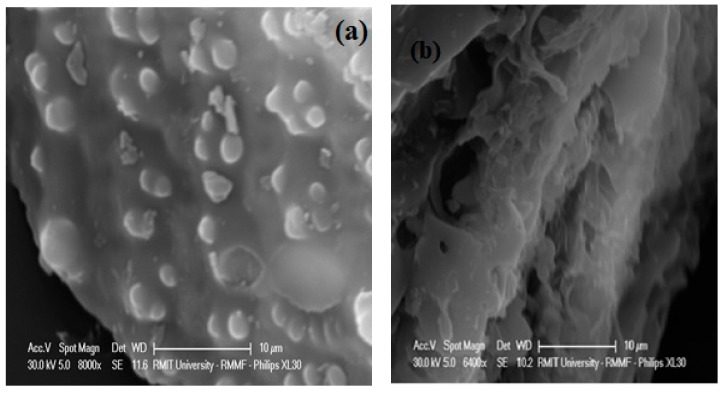
The SEM of (**a**) the rice straw and (**b**) the optimized hydrochar.

SEM is one of the techniques used to analyse the morphological structures of biomass and the products obtained from biomass by various processes. The SEM images of rice straw and optimized hydrochars are shown in Figure 7a,b. As expected, the morphological structure of the hydrochar has changed by forming pores on its surface, suggesting that the structural transformation of rice straw occurred during the MIHTC. This behavior implies that the lignocellulosic structure of rice straw was eroded during the MIHTC.

It is evident from Figure 7a that there are no pores available on the surface of rice straw, while the pores were formed on the hydrochar surface due to the removal of volatile matter during the MIHTC process [22]. The pore formations on the hydrochar surfaces are influenced by the reaction temperature and time of MIHTC; hemicellulose and cellulose decompose significantly at higher reaction times, causing the formation of pores throughout the hydrochar [60]. The biomass is significantly degraded at higher temperatures. The higher temperatures cause an exposure of layers of the biomass improving the hydrochar porosity. Marx et al. [64] suggests that the walls of neighboring pores are destroyed at higher temperatures resulting in a broadening of the pore diameters. The pore structures on the hydrochar surfaces allow dewatering of the hydrochar more easily during filtration as the hydrochar pores enable water drainage from the MIHTC product. Such properties also support the rapid drying of hydrochars [22]. The porous structure of hydrochars produced at higher temperatures of the MIHTC means that the hydrochar can potentially be utilized for adsorption and sequestration applications [14].

Although the porosity of hydrochar is improved after MIHTC and it is greater than in the raw rice straw, it is well documented in the literature that the porosity of hydrochars is quite low and not well upgraded during HTC due to the deposition of secondary microspheres on the top of the primary chars. Therefore, the activation of hydrochars (either physical or chemical) is necessary to get a higher porosity and to improve the hydrochar adsorption capacity [65].

#### 3.2.6. XRD Analysis

**Figure 8:**
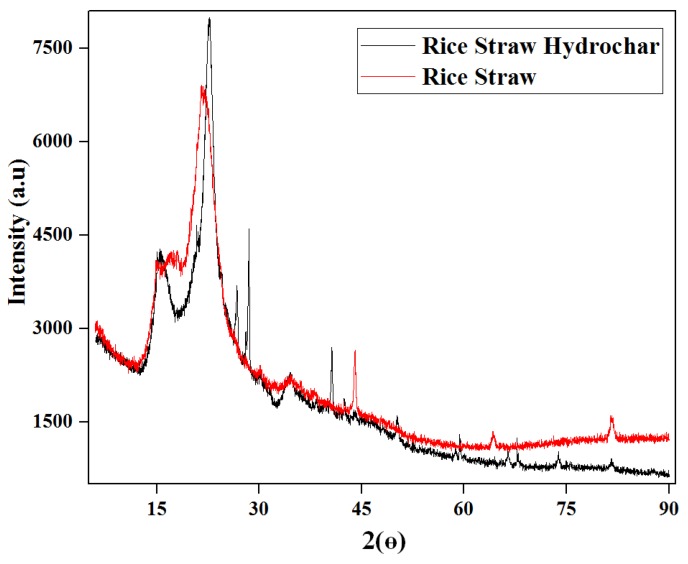
The XRD analysis of rice straw and the optimized hydrochar.

The XRD pattern displaying the crystallinity of the rice straw and optimized hydrochar is shown in Figure 8. Typically, two characteristic peaks were observed at 2θ of 15° and 26°, which are reported to be typical cellulose peaks [66]. The increased intensity of peaks is located in the range of 10° and 35° (2θ), which is recognized to be due to the diffraction of amorphous carbon [40]; this indicates that the rice straw was successfully carbonized to a carbon form. The crystallinity percentage of hydrochar was improved during MIHTC, which is due to the loss of the amorphous components, i.e., lignin and hemicellulose [67], existing in rice straw. According to Liu et al. [68], a higher crystallinity of hydrochar is credited to the hydrolysis of hemicellulose and cellulose in the amorphous region during the hydrothermal processing. It also was reported that crystallinity is indirectly proportional to the reaction temperature, suggesting that crystallinity will decrease by increasing the temperature.

#### 3.2.7. TGA/DTG Analysis

**Figure 9:**
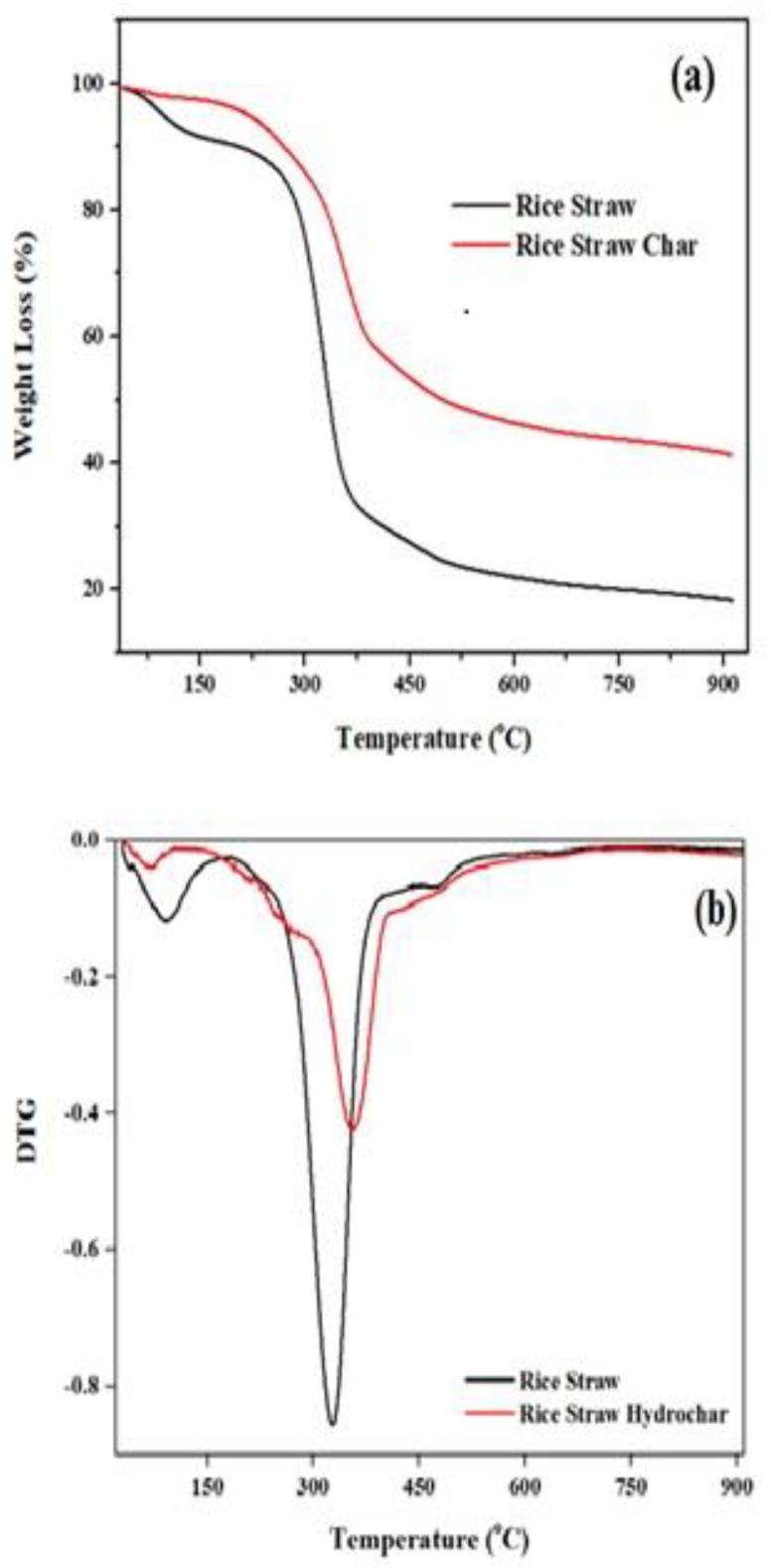
(**a**) The TGA analysis and (**b**) DTG analysis of rice straw and optimized hydrochars.

In this study, TGA was used to study the thermal stability of the raw rice straw and optimized hydrochars in order to study the thermal stability of the materials. Figure 9a,b shows the TGA and DTG analysis of the raw rice straw and the optimized hydrochar. It is observed from Figure 9a that in the first stage, moisture was removed up to 150 °C from both the samples due to evaporation and dehydration processes [19]. The second stage of weight loss occurred between 220–380 °C, which is due to the decomposition of hemicellulose and cellulose occurring at these temperatures. Elaigwu and Greenway [17] suggested that weight loss taking place between 220 °C and 410 °C is attributed to hemicellulose and cellulose decomposition whereas chemical bond breakage is initiated to release light volatile compounds. The low rate of mass loss after 400 °C indicates the level of nonvolatile matter, with the optimized char having a much higher level of this material.

The DTG graph shows two peaks for both the materials (refer Figure 9b). The first peak, which is in drying region, was at 50–150 °C which is attributed to the dehydration of the rice straw and hydrochar [69]. The second peak was noted at 300–350 °C, which is a sharp and tall peak, and it is due to the degradation of hemicellulose and cellulose [70]. The peaks denote that weight loss of hydrochar by hemicellulose and cellulose degradation is much lower as much of these components have been altered by the MIHTC process [71].

#### 3.2.8. N_2_ Adsorption/Desorption Isotherm

**Figure 10:**
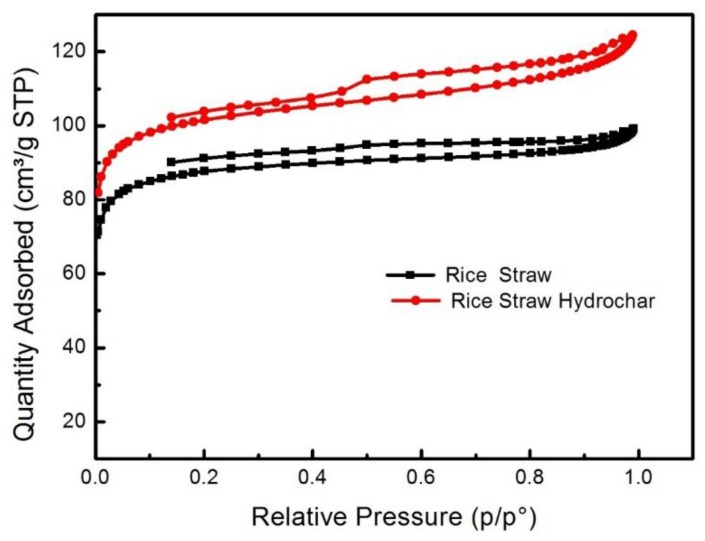
The N_2_ adsorption iotherm of rice straw and optimized hydrochars.

The N_2_ adsorption isotherm for rice straw and optimized hydrochar is shown in Figure 10. The hysteresis loop behavior indicates the adsorption fits the type I and type IV isotherms. The N_2_ adsorption behavior of the optimized hydrochar is greater than that of the rice straw, which is due to a rise in the relative pressure of N_2_ molecules which are located in wider pores, making a greater adsorption and demonstrating the presence of micropores and mesopores.

The N_2_ adsorption at low relative pressures, i.e., P/P_o_ < 0.1, indicates that new micropores were formed in large quantities because of the MIHTC of rice straw, which supported new pore formation. A rise in N_2_ uptake at high relative pressures, i.e., P/P_o_ > 0.2, is attributed to the development of micropores and mesopores. Moreover, N_2_ adsorption went up steadily at high relative pressures, exhibiting a higher volume of micropores and presence of small mesopores.

## 4. Conclusions

The effect of the process parameters of MIHTC including temperature, time, particle size and biomass to water ratio was optimized using the CCD methodology. The optimal yield of hydrochars was obtained at a 180 °C reaction temperature, a 20 min reaction time, a 1:15 w/v biomass to water ratio and a 3 mm particle size. The results suggest that the transformation of rice straw occurred during the MIHTC due to different reactions taking place including decarboxylation, dehydration and demethylation, which led to the improvement in energy and the chemical and structural characteristics of the optimized hydrochar. The HHV, carbon content and fixed carbon values increased whereas the oxygen content, moisture and volatile matter contents decreased in the hydrochar. These values were similar to various types of coal, confirming the potential suitability of hydrochar as a solid fuel. The SEM results suggest that the fibrous structure of rice straw was eroded during MIHTC, which caused pores formation on the hydrochar surface. The TGA/DTG result shows that thermal stability of the hydrochar was enhanced after MIHTC.

Based on the results of the HHV, energy density, ultimate analysis and proximate analysis, it can be suggested that the hydrochar can potentially be utilized as a solid fuel comparable to different coals. Other findings also suggest that the potential applications of hydrochar could include adsorption, carbon sequestration, energy and agriculture applications.

## Figures and Tables

**Table 1:** The process variables with their levels for synthesis of hydrochar via microwave-induced hydrothermal carbonization (MIHTC).

Factor	Unit	High Coded	Low Coded	High	Low
Temperature (A)	°C	1	−1	220	180
Time (B)	min	1	−1	60	20
Particle Size (C)	mm	1	−1	3	1
Biomass to Water Ratio (D)	w/v	1	−1	1:15	1:5

**Table 2:** The experimental design matrix suggested by the central composite design (CCD) with the corresponding response results of the hydrochar yield (wt %).

Std	Run	Factor (A)	Factor (B)	Factor (C)	Factor (D)	Response (Yield%)
24	1	220	20	1	1:5	40.1
5	2	180	60	2	1:15	55.1
10	3	220	20	1	1:5	39.55
21	4	180	40	3	1:5	55.5
8	5	180	40	3	1:5	55.43
14	6	220	20	3	1:5	40.56
15	7	200	40	1	1:15	44.63
16	8	220	60	1	1:5	38.2
13	9	200	20	2	1:5	45.74
18	10	200	40	3	1:15	45.5
17	11	200	40	2	1:5	45.12
12	12	180	20	1	1:10	56.7
3	13	180	60	1	1:5	54.8
11	14	220	40	2	1:10	38.9
19	15	200	20	3	1:10	46.86
1	16	220	60	3	1:5	38.56
6	17	220	20	1	1:15	39.98
7	18	180	20	3	1:15	57.89
9	19	220	60	3	1:15	38.12
25	20	180	60	2	1:15	54.93
22	21	200	20	2	1:5	46.73
23	22	220	20	1	1:15	40.21
4	23	200	60	3	1:10	42.6
2	24	220	60	1	1:15	37.95
20	25	220	20	3	1:15	40.45

**Table 3:** The ANOVA for the hydrochar yield % of the MIHTC of rice straw.

Source	Sum of Squares	DF	Mean Square	F Value	Prob > F	Status
Model	1184.857	14	84.63266	396.0004	<0.0001	significant
(A) Temperature	1032.732	1	1032.732	4832.204	<0.0001	
(B) Time	23.87823	1	23.87823	111.7274	<0.0001	
(C) Particle Size	0.148824	1	0.148824	0.696356	0.4235	
(D) Water to Biomass	0.291942	1	0.291942	1.366012	0.2696	
AB	0.350942	1	0.350942	1.642077	0.2290	
AC	0.180202	1	0.180202	0.843175	0.3801	
AD	0.327317	1	0.327317	1.531533	0.2442	
BC	0.645949	1	0.645949	3.022428	0.1128	
BD	0.202082	1	0.202082	0.945552	0.3538	
CD	0.00162	1	0.00162	0.007578	0.9323	
A^2^	24.85305	1	24.85305	116.2886	<0.0001	
B^2^	0.054034	1	0.054034	0.252827	0.6260	
C^2^	0.210648	1	0.210648	0.985632	0.3442	
D^2^	0.583457	1	0.583457	2.730023	0.1295	
Residual	2.137186	10	0.213719			
Lack of Fit	1.452536	5	0.290507	2.121575	0.2143	not significant
Pure Error	0.68465	5	0.13693			
Cor Total	1186.994	24				

**Table 4:** The higher heating valuse (HHV) (MJ/kg) of rice straw, the optimized hydrochar, the different hydrochars synthesised by MIHTC and various coal types.

Materials	HHV (MJ/kg)	References
Rice Straw	12.3	This Study
Rice Straw Hydrochar	17.6	This Study
Bamboo Hydrochar	17.2	[30]
Barley Straw Hydrochar	17.8	[31]
Wheat Straw Hydrochar	17.9	[31]
Rapeseed Husk Hydrochar	21.6	[21]
Lignite Coal	16.9	[32]
Peat	15.4	[32]

**Table 5:** The proximate analysis of rice straw, the optimized hydrochar and different types of coals.

Materials	Moisture (%)	Fixed Carbon (%)	Volatile Matters (%)	Ash (%)	References
Rice Straw	8.53	14.37	70.20	6.9	This study
Rice Straw Hydrochar	2.5	35.4	45.6	16.5	This study
Australian Bituminous Coal	3.8	33.0	39.6	23.6	[54]
Indonesian Subbituminous Coal	-	46.8	50.2	28.7	[55]
Chinese Lignite Coal	8.1	26.7	50.1	23.2	[56]
Chinese Bituminous Coal	13.5	59.7	31.4	8.9	[56]
South African Coal	-	56.1	25.8	18.0	[57]
